# Single‐Cell Multiomics Reveals That OTUD7B Is a Central Driver of Epithelial Remodelling and the Immunosuppressive Microenvironment in Pancreatic Ductal Adenocarcinoma

**DOI:** 10.1111/jcmm.71322

**Published:** 2026-10-11

**Authors:** Xiao‐Li Liu, Jing‐Wang, Shan‐Yu Zhao, Lu‐Li, Ming‐Hui Zhang

**Affiliations:** ^1^ Department of Pathology General Hospital of Ningxia Medical University Yinchuan China; ^2^ Department of Pathology, School of Basic Medical Sciences Dali University Yunnan China

**Keywords:** OTUD7B, pancreatic ductal adenocarcinoma, tumour microenvironment

## Abstract

Pancreatic ductal adenocarcinoma (PAAD) is characterized by extreme malignancy and profound resistance to therapy, largely driven by a fibrotic and immunosuppressive tumour microenvironment (TME). The deubiquitinase OTU domain‐containing protein 7B (OTUD7B) has been implicated in inflammatory and oncogenic signalling, but its role in PAAD and its TME remains unclear. This study integrated single‐cell RNA‐seq datasets from the GEO and bulk RNA‐seq data from the TCGA to delineate the cellular ecosystem of PAAD, and employed cell–cell communication analysis, high‐dimensional weighted gene coexpression network analysis (hdWGCNA), and nonnegative matrix factorization (NMF) to identify OTUD7B‐associated modules and epithelial subpopulations, while assessing the survival and clinicopathologic correlations of OTUD7B in the TCGA‐PAAD cohort. Quantitative proteomics of OTUD7B‐knockout PAAD cells was performed and combined with GO/KEGG and GSEA to explore downstream pathways, and immune infiltration was inferred by deconvolution algorithms and validated by multiplex immunofluorescence staining of OTUD7B, CD4, and FOXP3. Single‐cell RNA‐seq revealed the expansion of epithelial cells, fibroblasts, and macrophages with dense ligand–receptor networks, alongside the depletion of effector T and NK cells, indicating a shift toward an immunosuppressive TME. hdWGCNA identified epithelial–ECM modules in which OTUD7B served as a hub gene, and NMF revealed an OTUD7B‐high epithelial subset enriched in glycosylation‐related metabolic programs and positioned at an early stage of the malignant trajectory. OTUD7B was significantly upregulated in PAAD and was associated with higher grade, lymph node metastasis, and advanced stage, and predicted poorer overall survival. Proteomic profiling of OTUD7B‐deficient cells revealed broad remodelling of mitochondrial oxidative phosphorylation, ROS metabolism, and redox homeostasis. OTUD7B expression was positively correlated with M2 macrophage infiltration, immunomodulatory gene expression, and CD4^+^FOXP3^+^ regulatory T‐cell accumulation, and tissue staining further confirmed the positive association between OTUD7B expression and Treg‐rich regions. Collectively, OTUD7B acts as a key integrative node that links epithelial remodelling, mitochondrial metabolic reprogramming, and TME immunosuppression in PAAD, serving as an adverse prognostic biomarker and a potential therapeutic target to remodel tumour metabolism and enhance antitumor immunity.

## 1 Introduction

Pancreatic ductal adenocarcinoma (PAAD) is among the most lethal solid malignancies and is characterized by insidious onset, early metastatic spread, and resistance to virtually all available therapies [1]. Despite advances in surgical techniques, chemotherapy, radiotherapy, and targeted agents, the 5‐year overall survival of patients with PAAD remains dismal [2]. Conventional genomic studies have revealed recurrent driver mutations such as those in *KRAS*, *TP53*, *CDKN2A*, and *SMAD4*; however, attempts to translate these alterations into effective therapeutic strategies have been largely unsuccessful. Accumulating evidence indicates that in addition to intrinsic genetic lesions, the highly complex and immunosuppressive tumour microenvironment (TME) plays a central role in driving tumour initiation, progression, and treatment failure in PAAD [2, 3].

The PAAD TME is notable for its dense desmoplastic stroma, which is composed of activated fibroblasts, abundant extracellular matrix, and aberrant vasculature and is interwoven with diverse immune cell populations. Rather than mounting effective antitumor immunity, these immune infiltrates are often skewed toward immunosuppressive phenotypes, including tumour‐associated macrophages, regulatory T cells (Tregs), and dysfunctional T cells, which collectively promote immune evasion [4]. Crosstalk among epithelial, stromal, and immune compartments—mediated by cytokines, chemokines, metabolic cues, and cell–cell contact—critically shapes this ecosystem [5]. However, the cellular architecture and communication networks that underlie PAAD progression, as well as the key molecular nodes that coordinate epithelial plasticity, stromal remodelling, and immune suppression, remain incompletely defined. Single‐cell transcriptomics and high‐dimensional proteomics now offer powerful means to deconvolute this complexity at unprecedented resolution.

OTU deubiquitinase 7B (OTUD7B, also known as Cezanne) is a member of the ovarian tumour (OTU) family of deubiquitinating enzymes and has emerged as an important regulator of inflammatory and oncogenic signalling pathways. By editing K63‐ and K11‐linked ubiquitin chains on specific substrates, OTUD7B can modulate NF‐κB, MAPK, and other signalling cascades that influence cell survival, proliferation, and immune responses [6]. Dysregulated OTUD7B expression has been reported in several tumour types, and accumulating data suggest context‐dependent roles in tumour biology and immune regulation [7]. Nevertheless, the expression pattern, functional significance, and mechanistic impact of OTUD7B within the unique microenvironment of PAAD are largely unknown, particularly with respect to how it may coordinate epithelial reprogramming, mitochondrial metabolism, and the immunosuppressive TME.

In this study, we combined single‐cell RNA sequencing, high‐dimensional weighted gene coexpression network analysis, nonnegative matrix factorization, bulk transcriptomic profiling, quantitative proteomics, and multiparameter immunofluorescence to systematically investigate the role of OTUD7B in PAAD. We first delineated the cellular landscape and intercellular communication networks of PAAD and normal pancreas, revealing profound remodelling of epithelial, stromal, and immune compartments. We then identified OTUD7B as a hub gene within epithelial and extracellular‐matrix–associated coexpression modules and defined an OTUD7B‐high epithelial subpopulation characterized by metabolic reprogramming. By integrating clinical cohorts, we demonstrated that OTUD7B is markedly upregulated in PAAD, is correlated with aggressive clinicopathological features, and is a predictor of poor overall survival. Mechanistically, proteomic and pathway analyses of OTUD7B‐deficient cells revealed extensive alterations in mitochondrial oxidative phosphorylation, redox homeostasis, and immune‐related signalling. Finally, immune infiltration analyses and tissue‐level validation revealed that high OTUD7B expression is closely associated with an immunosuppressive microenvironment enriched in Tregs and protumor myeloid cells. Together, these findings establish OTUD7B as a key orchestrator of epithelial remodelling and TME immunosuppression in PAAD and highlight it as a potential biomarker and therapeutic vulnerability.

## 2 Materials and Methods

### 2.1 Data Sources and Patient Cohorts

Single‐cell RNA sequencing (scRNA‐seq) data from pancreatic ductal adenocarcinoma (PAAD) and nontumor pancreatic tissues were obtained from publicly available databases (e.g., GEO/ArrayExpress), with matched raw count matrices and cell annotations where available. Bulk RNA‐seq data and corresponding clinicopathological information for the PAAD and pancancer cohorts were downloaded from The Cancer Genome Atlas (TCGA) and the GTEx project via the UCSC Xena platform.

### 2.2 Single‐Cell RNA‐Seq Preprocessing, Clustering and Trajectory Analysis

Raw scRNA‐seq data were processed using Seurat (v4.x) in R [8]. Cells with a low number of detected genes, high mitochondrial RNA content, or extreme UMI counts were removed to exclude low‐quality cells and doublets. Gene expression matrices were normalized using the LogNormalize method and scaled to regress out technical covariates (e.g., UMI counts and mitochondrial percentages). Batch effects across samples were corrected using canonical correlation analysis (CCA) or Harmony integration [9]. Principal component analysis (PCA) was performed on highly variable genes, and shared nearest neighbour (SNN) graphs were constructed based on the top principal components. Unsupervised clustering was carried out using the Louvain algorithm, and clusters were visualized by uniform manifold approximation and projection (UMAP) [10]. Cell types were annotated according to canonical marker genes for T cells, B cells, macrophages, monocytes, fibroblasts, endothelial cells, epithelial cells, smooth muscle cells, NK cells, tissue stem cells, neutrophils and neurons, supplemented by reference‐based annotation when necessary.

To characterize dynamic epithelial transitions, pseudotime trajectory analysis was performed on epithelial cells using Monocle3 or a similar trajectory inference tool [11]. After dimensionality reduction (UMAP/DDRTree) and graph learning, the cells were ordered along pseudotime, and genes whose expression patterns significantly changed were identified. The expression patterns of key genes, including OLFM4, ONECUT2, CDHR2 and OTUD7B, were examined along pseudotime to elucidate the transition from a normal‐like state to a malignant state.

### 2.3 Cell–Cell Communication and Gene Coexpression Network Analysis

High‐dimensional weighted gene coexpression network analysis (hdWGCNA) was performed on single‐cell data to identify gene modules associated with specific cell types and biological processes [12]. After highly variable genes were selected, an adjacency matrix was constructed using a soft‐threshold power chosen to approximate scale‐free topology. The topological overlap matrix (TOM) was computed and subjected to hierarchical clustering with dynamic tree cutting to define coexpression modules (MP1–MP9) [13]. Module eigengenes were correlated with cell type and epithelial state. Hub genes within modules were identified based on intramodular connectivity, and functional enrichment (e.g., ECM remodelling, immune regulation, and epithelial adhesion) was assessed using clusterProfiler for Gene Ontology and pathway analysis.

### 2.4 NMF‐Based Epithelial Subtyping

To resolve epithelial heterogeneity, nonnegative matrix factorization (NMF) was applied to the epithelial cell expression matrix using the NMF R package [14]. A range of factorization ranks (*k*) was tested, and the optimal rank was determined based on cophenetic correlation, dispersion and residual sum of squares. Each epithelial cell was assigned to an NMF‐derived metagene, generating distinct epithelial subsets. An OTUD7B^high subset^ (EPI‐OTUD7B‐C1) was identified, and its abundance was compared across samples. The functional enrichment of subset‐specific marker genes was performed using GO and KEGG, with particular attention given to glycosylation‐related metabolic pathways.

### 2.5 Bulk Transcriptomic and Pancancer Analyses

For the TCGA and GTEx data, the FPKM/TPM values were log2‐transformed after a small offset was added [15]. OTUD7B expression was compared between tumour and normal tissues across pancancer cohorts and specifically in PAAD using Wilcoxon rank‐sum or *t*‐tests, as appropriate. In the TCGA‐PAAD cohort, patients were stratified into high‐ and low‐OTUD7B groups according to the median or optimal cutoff determined by maximally selected rank statistics. Associations between OTUD7B expression and clinicopathological features (grade, stage, and lymph node status) were assessed using chi‐square tests or linear models. Kaplan–Meier survival curves and log‐rank tests, together with Cox proportional hazards models, were used to evaluate the prognostic value of OTUD7B for overall survival, and the results were validated in an independent PAAD cohort.

### 2.6 Proteomic Profiling After OTUD7B Knockout

Human PAAD cell lines with stable OTUD7B knockout were generated using the CRISPR/Cas9 system, and the corresponding control cells expressed a nontargeting sgRNA. Cells were lysed, and proteins were extracted, reduced, alkylated and digested with trypsin. Peptides were either labelled with tandem mass tags (TMTs) or analysed in a label‐free manner and then subjected to LC–MS/MS using a high‐resolution mass spectrometer. Raw spectra were processed with a standard proteomics pipeline (e.g., MaxQuant), and protein intensity values were normalized. Differentially expressed proteins between OTUD7B‐deficient and control cells were identified using moderated *t*‐tests with Benjamini–Hochberg correction. GO and KEGG enrichment analyses were performed on significantly altered proteins to identify pathways related to oxidative phosphorylation, the mitochondrial respiratory chain, the NADH dehydrogenase complex, redox homeostasis and ROS metabolism. Gene set enrichment analysis (GSEA) was further applied to ranked protein or transcript lists to confirm enrichment of mitochondrial and transmembrane transport pathways [16].

### 2.7 Tet‐On Doxycycline‐Inducible Stable Cell Lines

To generate a doxycycline‐inducible OTUD7B line, the open reading frame was PCR‐amplified from 293T cDNA and inserted into PLVX‐Tet‐On‐Puro. After stable transduction and selection with 2 μg mL^−1^ puromycin, 500 nM doxycycline was added for 36 h to trigger expression; empty‐vector, uninduced and induced populations were compared by Western blotting.

### 2.8 Cell Counting Kit‐8 (CCK‐8) Assay

For proliferation analysis, 3 × 10^3^ cells per well were seeded in 96‐well plates in triplicate. After adherence, 10 μL of CCK‐8 reagent was added at the indicated time points, and the plates were incubated at 37°C for 60 min. Absorbance at 450 nm was measured with a microplate reader and averaged across three independent runs.

### 2.9 Colony Formation Assay

To evaluate the long‐term proliferative capacity, stably transfected PAAD cells were plated at 1 × 10^3^ cells per well in 6‐well plates and cultured for 10 days in medium containing 10% FBS. Colonies were fixed with 4% paraformaldehyde (20 min), rinsed three times with PBS, and incubated with 0.1% crystal violet for 20 min. After three further washes, the plates were left inverted to dry at room temperature and then photographed for quantification. The relative clone density was calculated by normalizing the colony number of each experimental group to that of the control group, which was set as 1.

### 2.10 Wound‐Healing Migration Assay

Pancreatic cancer cells were seeded into 6‐well plates and cultured to 90% confluence. A linear scratch was made across the monolayer using the tip of a sterile 20‐μL pipette. Photographs were taken immediately (0 h) and after 24 or 48 h. Wound width was quantified using ImageJ software, and the percentage of wound healing was calculated based on three independent experiments.

### 2.11 Western Blot Analysis

Cells were lysed in RIPA buffer (Beyotime Institute of Biotechnology, Jiangsu, China) supplemented with protease inhibitor cocktail, and the protein content was quantified with a DC assay (Bio‐Rad). Thirty‐microgram aliquots were resolved by 10% SDS–PAGE, transferred to PVDF membranes, blocked with 5% skim milk in TBST, and probed sequentially with primary antibodies (4°C, overnight; OTUD7B and GAPDH 1:2000) and HRP‐conjugated secondary antibodies (1:8000, 1 h, RT). Bands were visualized by ECL and quantified using ImageJ.

### 2.12 Assessment of Malondialdehyde (MDA) and Superoxide Dismutase (SOD)

Intracellular levels of MDA and total SOD activity were measured using commercial colorimetric assay kits (Beyotime Institute of Biotechnology, Jiangsu, China) according to the manufacturer's instructions.

### 2.13 Measurement of Intracellular Reactive Oxygen Species (ROS)

Intracellular ROS levels were measured using ROS detection kit (Beyotime, China). The cells were seeded in 24‐well plates and incubated with 10 μM DCFH‐DA at 37°C for 20 min in the dark, followed by washing with PBS. Fluorescence intensity was detected by fluorescence microscopy. The mean fluorescence intensity (MFI) was quantified using ImageJ and used to represent ROS levels in each group.

### 2.14 Immunohistochemistry, Multiplex Immunofluorescence and Image Analysis

FFPE sections from PAAD and adjacent nontumor tissues were used for immunohistochemistry (IHC) and multiplex immunofluorescence. After deparaffinization and antigen retrieval, the sections were blocked and incubated with primary antibodies against OTUD7B and epithelial markers, followed by HRP‐conjugated secondary antibodies and chromogenic detection for IHC. For multiplex immunofluorescence, sequential or simultaneous staining was performed using antibodies against OTUD7B, CD4 and FOXP3 combined with fluorophore‐conjugated secondary antibodies and nuclear counterstaining (e.g., DAPI). Slides were scanned using a multispectral or fluorescence scanner, and quantitative image analysis was carried out using dedicated software. OTUD7B staining intensity, FOXP3 fluorescence, and the FOXP3/CD4 ratio were quantified in tumour and adjacent tissues. The colocalization of OTUD7B with CD4^+^FOXP3^+^ regulatory T cells was evaluated based on spatial overlap and correlation of fluorescence signals.

### 2.15 Immune Infiltration and Immunomodulatory Correlation Analysis

Immune cell infiltration in bulk PAAD samples was estimated using CIBERSORT, TIMER or similar deconvolution algorithms based on normalized gene expression matrices [17, 18]. Proportions of major immune cell types, including M2 macrophages, CD8^+^ T cells, NK cells, dendritic cells and CD4 memory T cells, were inferred and correlated with OTUD7B expression using Pearson or Spearman methods. A curated panel of immunomodulatory genes, encompassing costimulatory and coinhibitory checkpoints, chemokines and chemokine receptors, was constructed, and correlation matrices between OTUD7B and these genes were constructed and visualized as heatmaps and scatterplots to delineate the OTUD7B‐associated immunoregulatory landscape.

Protein expression matrices derived from proteomics data were preprocessed and subjected to CIBERSORT analysis. Samples were stratified according to OTUD7B status (sgOTUD7B vs. parental), and differences in immune cell fractions between groups were systematically compared. Statistical significance was evaluated using appropriate methods, and the results were visualized to characterize the immune landscape associated with OTUD7B depletion.

### 2.16 Statistical Analysis

All the statistical analyses were performed in R (v4.x). Continuous variables were compared using Student's *t*‐test or the Wilcoxon rank‐sum test, and categorical variables were compared using the chi‐square or Fisher's exact test. Correlations were evaluated using Pearson or Spearman coefficients. Survival curves were generated using the Kaplan–Meier method and compared by log‐rank tests; hazard ratios and 95% confidence intervals were estimated using Cox proportional hazards models. Multiple testing was corrected using the Benjamini–Hochberg false discovery rate (FDR) procedure. Unless otherwise specified, a two‐sided *p* < 0.05 was considered to indicate statistical significance.

## 3 Results

### 3.1 Cellular Landscape and Cell–Cell Communication in the PAAD Microenvironment

To dissect the differences in cellular composition and the immune microenvironment between pancreatic ductal adenocarcinoma (PAAD) and normal pancreatic tissues, we first integrated and clustered single‐cell transcriptomic datasets. UMAP visualization separated all the cells into twelve major lineages, namely, T cells, B cells, macrophages, monocytes, fibroblasts, endothelial cells, epithelial cells, smooth muscle cells, NK cells, tissue stem cells, neutrophils and neurons, highlighting the pronounced cellular heterogeneity of pancreatic tissue (Figure 1A). Based on curated ligand–receptor pairs, we constructed a cell–cell communication network, which revealed that macrophages, fibroblasts and epithelial cells occupy central hubs within the tumour microenvironment and maintain extensive crosstalk with multiple lineages, implicating them as key regulators of immune responses and stromal remodelling in PAAD (Figure 1B). Quantitative analysis of interaction number and strength revealed markedly enhanced intercellular communication in tumour versus normal tissues, particularly between macrophages and fibroblasts and between these stromal cells and immune lineages, together with strengthened epithelial–immune interactions, collectively indicating a tumour‐associated immunosuppressive communication pattern (Figure 1C). Cell composition analysis further demonstrated that although T cells constituted the largest population overall, the relative proportions of epithelial cells, fibroblasts and macrophages were significantly increased in the PAAD samples, whereas the proportions of T cells and NK cells were decreased. This shift from immune‐cell dominance toward stromal and epithelial expansion suggests a transition of the tumour microenvironment from an immune‐active state to an immune‐suppressive state that favours tumour progression (Figure 1D).

**FIGURE 1:**
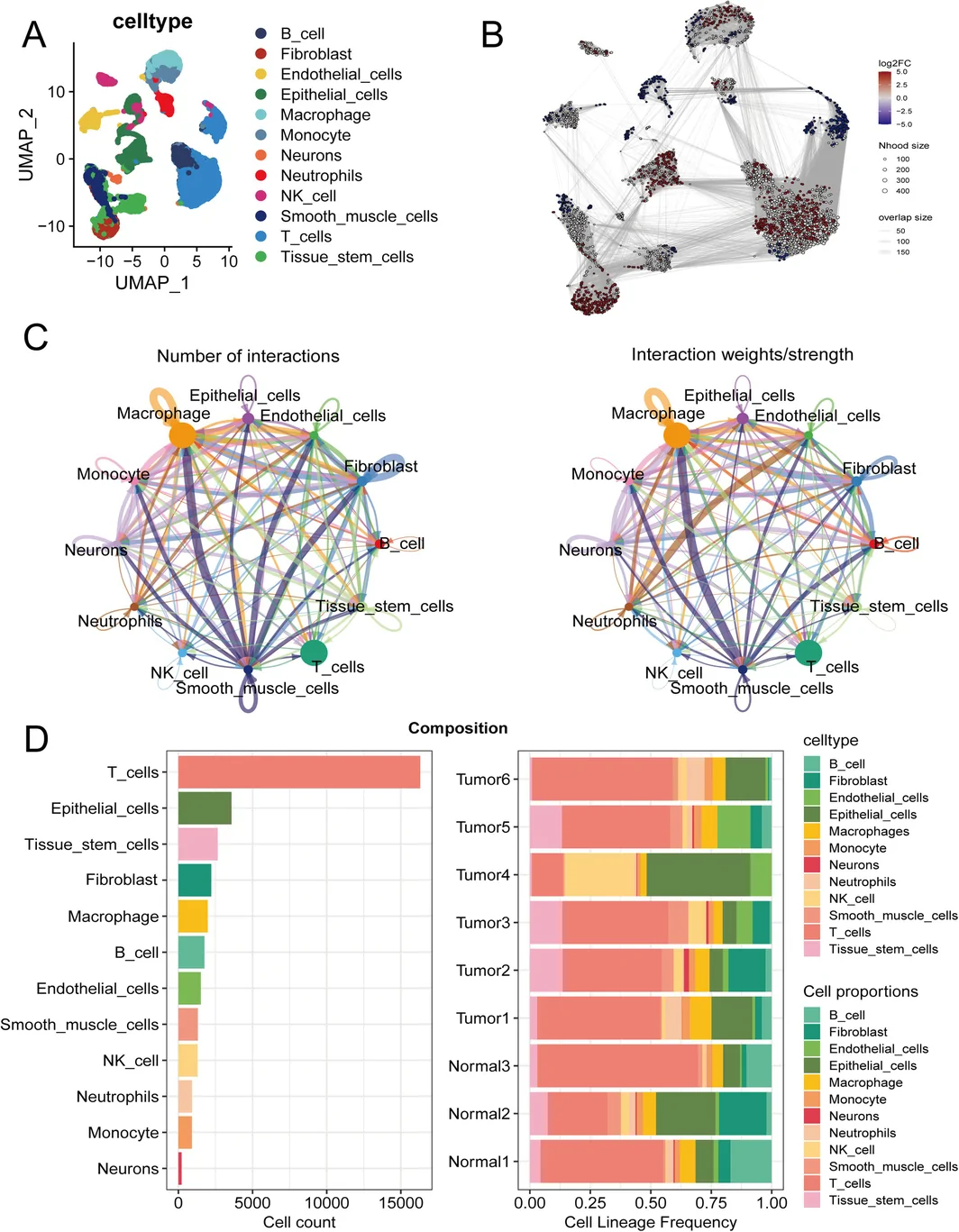
Single‐cell landscape and cell–cell communication in PAAD and normal pancreas. (A) UMAP plot showing 12 major cell types identified from integrated single‐cell RNA‐seq data. (B) Cell–cell interaction network highlighting communication among different cell populations. (C) Circle plots showing the number (left) and strength (right) of interactions between each cell type. (D) Bar plots displaying overall cell counts (left) and the proportions of each cell type across tumour and normal samples (right).

### 3.2 Cell Type‐Specific Gene Signatures and Malignant Transition of Epithelial Cells in PAAD

To further delineate the molecular characteristics and functional heterogeneity of the identified cell populations, we systematically analysed cell type–specific gene expression patterns and pathway enrichment profiles. As shown in Figure 2A, distinct marker gene signatures were observed across major cell types. Macrophages were characterized by high expression of C1QC and MS4A4A, fibroblasts by COL1A1 and COL3A1, epithelial cells by KRT19 and EPCAM, and T cells by GZMK and TRAT1, consistent with their respective roles in immune regulation, extracellular matrix remodelling, epithelial structure maintenance, and cytotoxic function.

**FIGURE 2:**
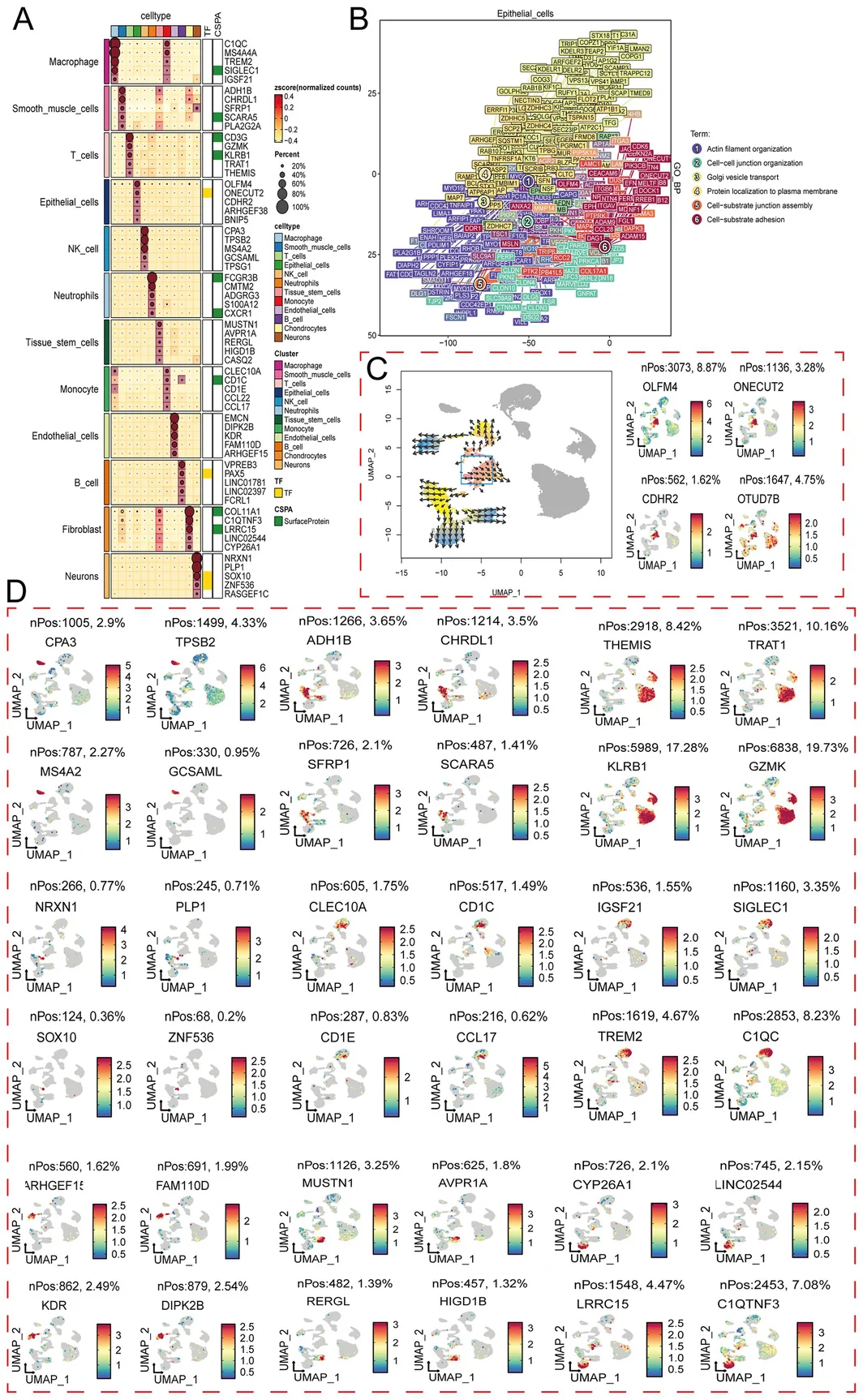
Cell‐type marker genes and functional features in the PAAD single‐cell data. (A) Heatmap showing canonical marker genes across major cell types. (B) GO enrichment map highlighting key biological processes in epithelial cells. (C) Pseudotime trajectory of epithelial cells with expression patterns of OLFM4, ONECUT2, CDHR2 and OTUD7B. (D) UMAP feature plots of representative differentially expressed genes across immune, stromal and epithelial lineages.

Gene set enrichment analysis of epithelial cells (Figure 2B) revealed significant enrichment in pathways associated with cytoskeletal organization, cell–cell junctions, and cell–matrix adhesion, suggesting enhanced cellular plasticity and invasive potential. Pseudotime trajectory analysis further demonstrated a continuous transition of epithelial cells from normal‐like to malignant states (Figure 2C), accompanied by dynamic changes in the expression of genes such as OLFM4, ONECUT2, CDHR2, and OTUD7B, implicating these factors in malignant progression and the maintenance of tumour cell stemness.

In addition, UMAP visualization of representative differentially expressed genes (Figure 2D), including immune‐related markers (TREM2, CLEC10A), tumour‐associated genes (TPSB2, ADH1B), and stromal remodelling factors (COL1A1, LRRC15), highlighted pronounced transcriptional and spatial heterogeneity within the tumour microenvironment. Collectively, these multilayered molecular features underscore the complexity and plasticity of the PAAD ecosystem.

### 3.3 Co‐Expression Modules Linking Epithelial Remodelling and Stromal Activation Centered on OTUD7B

To identify coordinated gene programs associated with specific biological processes in PAAD, we next performed high‐dimensional weighted gene coexpression network analysis (hdWGCNA) on the single‐cell transcriptomic data. The resulting network, in which all genes are grouped into multiple coexpression modules (MP1–MP9) characterized by strong intramodule correlations, is shown in Figure 3A, suggesting that the functional relevance of each module is shared. The soft‐thresholding analysis is shown in Figure 3B, where an optimal power of 8 was selected to approximate a scale‐free topology while maintaining adequate mean connectivity, providing a robust foundation for module detection. The gene clustering dendrogram is shown in Figure 3C, with distinct colours representing different modules; the clear separation between modules reflects good independence and functional specificity. The results of the hub‐gene analysis for each module are summarized in Figure 3D, revealing prominent functional themes: the brown module is enriched for extracellular‐matrix remodelling genes (including *COL1A1*, *COL3A1*, *FN1* and *OTUD7B*), the yellow module is enriched for immune‐regulatory chemokines (such as *CCL2*, *CXCL8* and *CXCL3*), and the red module contains multiple epithelial‐associated genes (including *EPCAM*, *KRT19* and *CDH1*), indicating that these modules are involved in tumour cell adhesion and migration. The module expression across epithelial cells is compared in Figure 3E, which shows that the turquoise, brown, blue and red modules are preferentially upregulated in tumour epithelial cells, indicating that their core genes may drive PAAD progression. The module–module correlation relationships are shown in Figure 3F, with several strong positive and negative associations; notably, the red and brown modules are highly positively correlated, suggesting coordinated epithelial transformation and stromal remodelling. The module eigengene scores across cell types are shown in Figure 3G, with the brown module and related MP modules peaking in epithelial cells. The shared gene *OTUD7B* at the intersection of these epithelial‐ and matrix‐associated modules points to a central role for OTUD7B in linking epithelial plasticity with extracellular matrix remodelling in PAAD.

**FIGURE 3:**
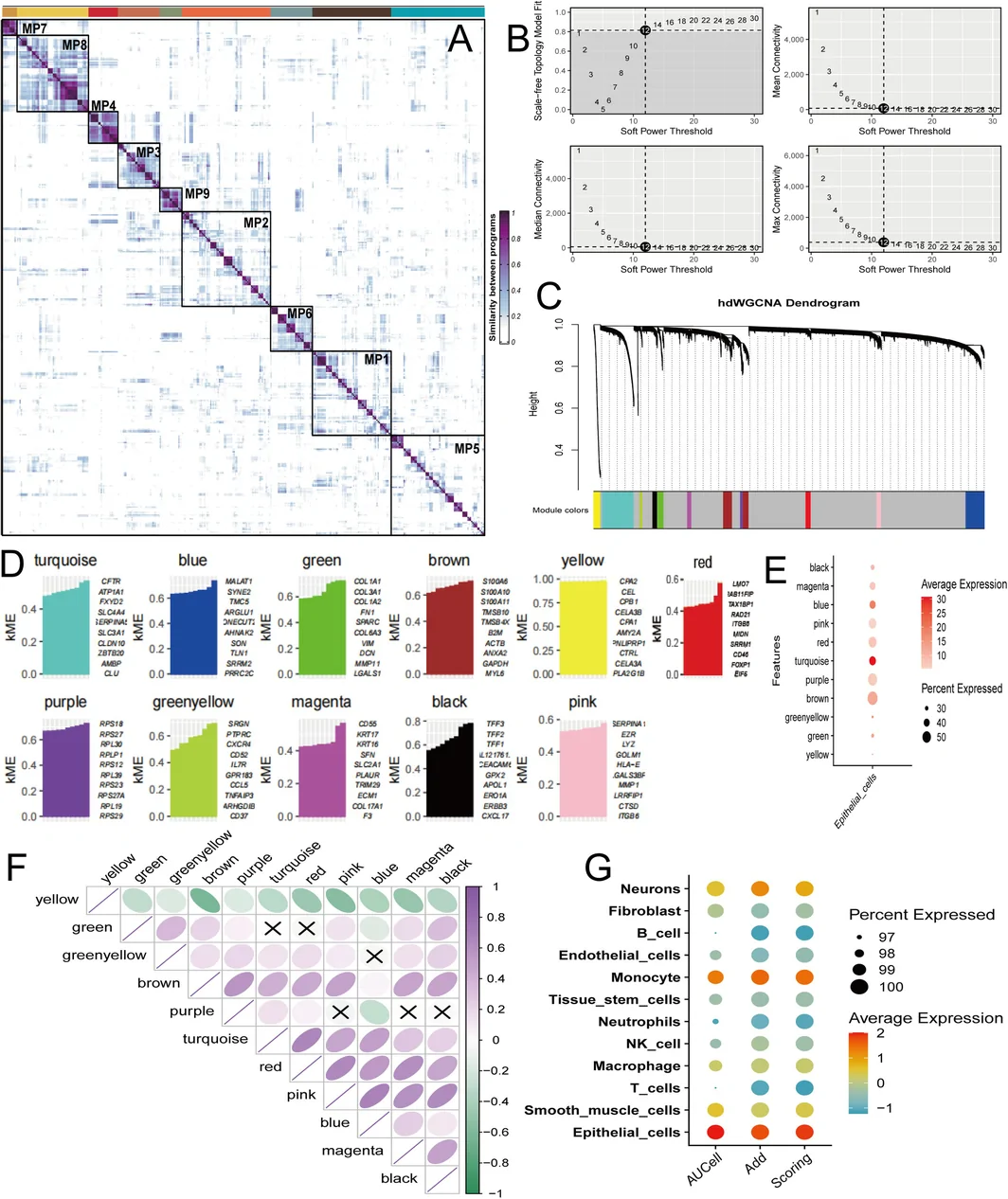
Coexpression modules identified by hdWGCNA in PAAD. (A) Topological overlap heatmap showing major gene coexpression modules (MP1–MP9). (B) Plots for soft‐threshold power selection ensuring scale‐free topology and adequate connectivity. (C) Gene clustering dendrogram with colour bars indicating assigned hdWGCNA modules. (D) Bar plots displaying representative hub genes and their module eigengene‐based connectivity in each module. (E) Dot plot summarizing the average expression and proportion of expressed genes for each module. (F) Correlation matrix of module eigengenes illustrating positive and negative relationships among modules. (G) Dot plot of module eigengene scores across major cell types, highlighting modules most enriched in epithelial cells.

### 3.4 OTUD7B‐Driven Epithelial Cell Subpopulations and Metabolic Reprogramming Revealed by NMF

To refine the heterogeneity of tumour epithelial cells and pinpoint OTUD7B‐driven subpopulations, we applied nonnegative matrix factorization (NMF) to the epithelial cell transcriptomes. This analysis revealed five distinct epithelial subsets, among which the EPI‐OTUD7B‐C1 cluster displayed the greatest abundance in PAAD (Figure 4A,B). Developmental trajectory analysis of these subsets revealed that OTUD7B expression peaks at an early stage during the malignant trajectory, suggesting that OTUD7B may participate in the initiation of epithelial transformation (Figure 4C,D). Functional enrichment analysis further revealed that the OTUD7B‐high epithelial subset is significantly associated with glycosylation‐related metabolic processes and other biosynthetic pathways, suggesting that OTUD7B‐driven cells undergo metabolic reprogramming that could support tumour growth and adaptation during PAAD development (Figure 4E).

**FIGURE 4:**
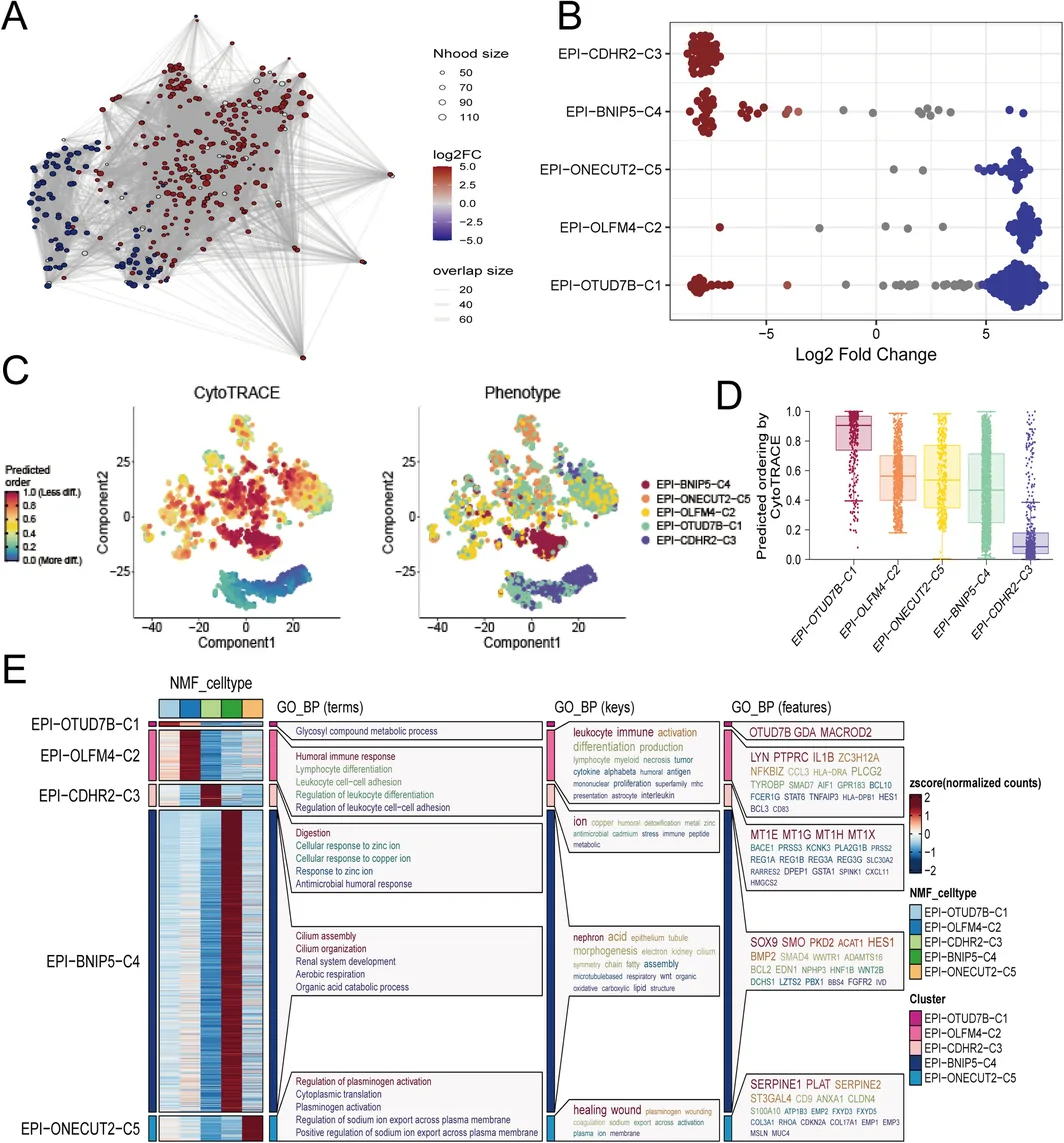
NMF‐defined epithelial subclusters and their functional features in PAAD. (A) NicheNet‐based interaction network highlighting epithelial neighbourhoods and their differential activity. (B) Scatter plot showing log2 fold changes in the expression of marker genes across five NMF‐derived epithelial subclusters (EPI‐OTUD7B‐C1, EPI‐OLFM4‐C2, EPI‐CDHR2‐C3, EPI‐BNIP5‐C4, and EPI‐ONECUT2‐C5). (C) CytoTRACE and phenotype maps depicting differentiation status and subcluster distribution along the epithelial trajectory. (D) Boxplots of the CytoTRACE scores for each epithelial subcluster, indicating relative stemness/immaturity. (E) Heatmap and GO biological process enrichment summarizing key functional programs of each epithelial subcluster, with representative genes highlighted.

### 3.5 OTUD7B Overexpression Pattern, Clinicopathological Association and Prognostic Significance in PAAD

Given the strong transcriptional and modular association of OTUD7B with malignant epithelial programs, we next evaluated its expression pattern and clinical significance in PAAD. Pancancer analysis revealed that OTUD7B is upregulated across multiple solid tumours, with particularly high levels in PAAD (Figure 5A). This upregulation was confirmed in the TCGA‐PAAD cohort, in which tumour tissues exhibited significantly higher OTUD7B mRNA expression than normal pancreas tissues did (Figure 5B). Immunofluorescence and immunohistochemistry further confirmed prominent OTUD7B protein expression predominantly in tumour epithelial compartments, which is consistent with the epithelial origin of PAAD (Figure 5C,D). Quantitative assessment demonstrated markedly higher staining intensity in tumour tissues than in adjacent nontumor tissues (Figure 5E). Moreover, OTUD7B expression increased with tumour grade and was significantly elevated in patients with lymph node metastasis (Figure 5F,G), suggesting a link to aggressive clinicopathologic behaviour. When patients were stratified according to OTUD7B expression, those with high OTUD7B levels tended to present with higher grades and more advanced clinical stages (Figure 5H). Kaplan–Meier survival analyses in both the TCGA cohort and an independent validation cohort consistently revealed that high OTUD7B expression is associated with poorer overall survival (Figure 5I), indicating that OTUD7B is a robust adverse prognostic biomarker in PAAD.

**FIGURE 5:**
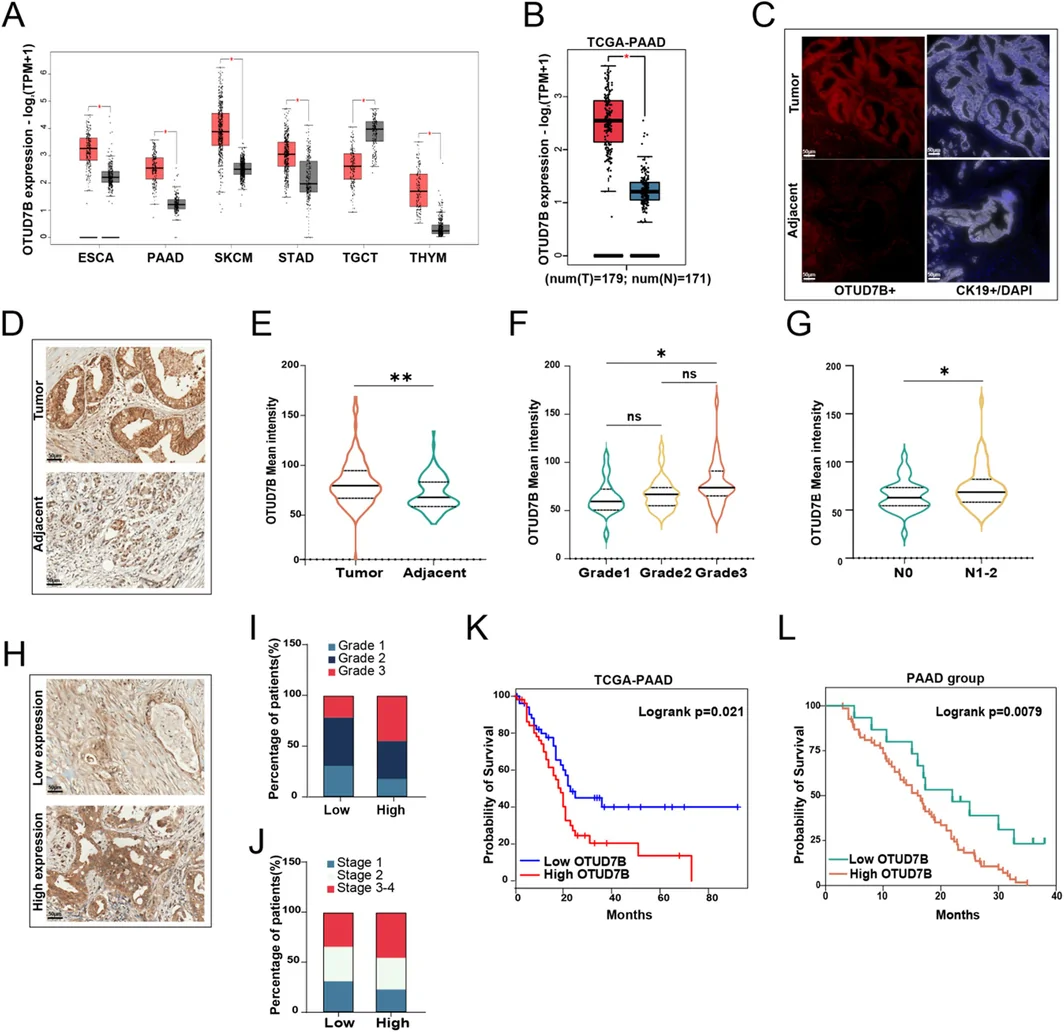
OTUD7B is overexpressed in PAAD and is associated with aggressive features and poor prognosis. (A) Pancancer comparison of the upregulation of OTUD7B expression in PAAD. (B) Boxplot of OTUD7B mRNA levels in TCGA‐PAAD tumours versus normal pancreas tumours. (C) Representative immunofluorescence images of OTUD7B and CK19 staining in tumour and adjacent tissues. Scale bar: 50 μm. (D) Representative immunohistochemistry of OTUD7B in PAAD and paired adjacent tissues. Scale bar: 50 μm. (E) Violin plot quantifying staining intensity between tumour and adjacent samples. (F) Violin plot showing OTUD7B expression across different histological grades. (G) Violin plot comparing OTUD7B expression in patients with or without lymph node metastasis. (H) Representative IHC images of low versus high OTUD7B expression in PAAD. Scale bar: 50 μm. Stacked bar charts illustrating the distribution of tumour grade (I) and clinical stage (J) between OTUD7B low‐ and high‐expression groups. (K, L) Kaplan–Meier survival curves in the TCGA‐PAAD cohort and an independent PAAD cohort stratified by OTUD7B expression. The * symbols in Figure 5A–B represent significant difference of OTUD7B protein expression between tumor and normal tissues in indicated cancer types in TCGA. The * in Figure 5F–G represent significant difference of OTUD7B protein expression between clinical pathological groups (Grade1 vs Grade3 groups; group with lymph node metastasis vs non‐metastasis group).

To systematically elucidate the role of OTUD7B in pancreatic cancer aggressiveness, we generated isogenic cell lines in which OTUD7B was either deleted via CRISPR/Cas9 or robustly overexpressed (Figure 6A–D). Clonogenic and CCK‐8 assays revealed that the loss of OTUD7B almost completely abolished anchorage‐dependent growth, whereas its enforced expression accelerated proliferation approximately twofold (Figure 6E–J). In wound‐healing assays, OTUD7B knockout markedly retarded gap closure, whereas its overexpression expedited migration to near‐complete wound resolution (Figure 6K–N). Collectively, these data establish OTUD7B as a critical driver of both proliferation and migration/invasion in pancreatic cancer cells.

**FIGURE 6:**
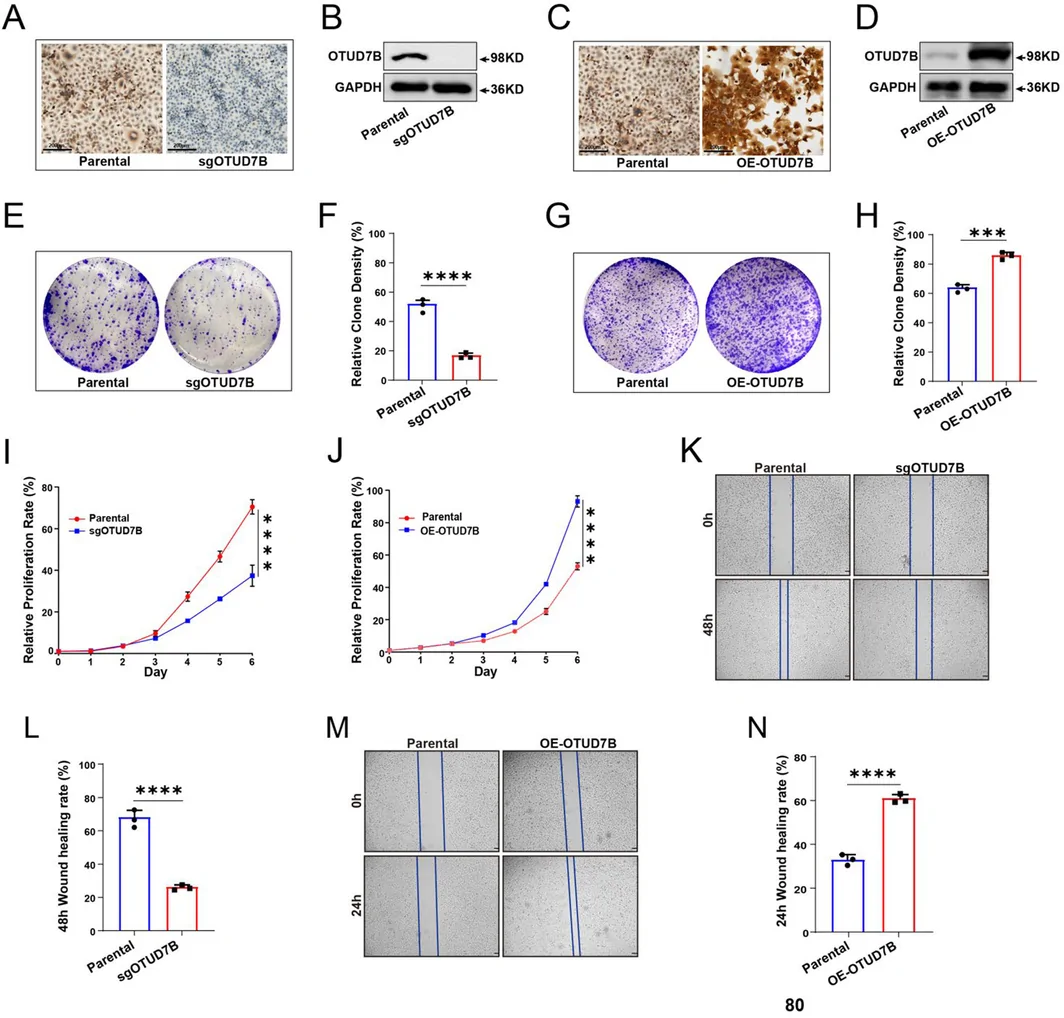
OTUD7B promotes the proliferation and migration of PANC‐1 cells in vitro. (A) Immunocytochemical staining of OTUD7B in parental and sgOTUD7B cells. Scale bar: 200 μm. (B) Validation of OTUD7B knockdown efficiency in parental vs. sgOTUD7B cells by Western blot. (C) Immunocytochemical staining of OTUD7B in parental and OE‐OTUD7B cells. Scale bar: 200 μm. (D) Validation of OTUD7B overexpression efficiency in parental vs. OE‐OTUD7B cells by Western blot. (E–H) Knockdown of OTUD7B significantly impaired colony‐forming capacity; overexpression of OTUD7B markedly enhanced colony formation. Relative clone density was normalized to the parental cell group. (I, J) CCK‐8 assays revealed that sgOTUD7B slowed cell proliferation, whereas OE‐OTUD7B accelerated it. (K–N) Wound‐healing capacity was strongly impaired in sgOTUD7B cells, whereas the scratch closure capacity of OE‐OTUD7B cells was significantly accelerated. Scale bar: 100 μm. The asterisks (****/***) indicate that OTUD7B overexpression or knockdown exerts statistically significant effects on the clonogenic capacity(6F,6H), proliferative activity(6I,6J), and migratory ability of tumor cells in vitro(6L,6N), as compared to the corresponding control groups.

### 3.6 Proteomic Remodelling After OTUD7B Knockout Highlights Mitochondrial and Oxidative Stress Pathways

To explore the mechanism underlying OTUD7B‐mediated oncogenic effects within the complex PAAD microenvironment, we conducted quantitative proteomic profiling in OTUD7B‐knockout pancreatic cancer cells. The heatmap in Figure 7A illustrates global proteomic remodelling upon OTUD7B loss, with widespread changes in proteins related to metabolism, inflammation and intracellular signalling. The volcano plot in Figure 7B highlights significantly upregulated and downregulated proteins, indicating that OTUD7B exerts broad regulatory control over cellular homeostasis. GO annotation and enrichment analyses further clarified these effects: Figure 7C shows that OTUD7B deficiency impacts pathways associated with oxidative phosphorylation, mitochondrial metabolism, cell adhesion and immune‐related functions. The GO enrichment plot in Figure 7D emphasizes the strong enrichment of processes such as NADH dehydrogenase complex assembly, mitochondrial respiratory chain organization, redox homeostasis and reactive oxygen species (ROS) metabolism, underscoring the central role of OTUD7B in mitochondrial function and oxidative stress regulation. The Sankey diagram and bubble plot in Figure 7E integrate the affected proteins into pathway networks, revealing that multiple components of mitochondrial complexes (e.g., NDUFS family members) converge on oxidative phosphorylation, metabolic regulation, thermogenesis and immune‐associated signalling. These results suggest that OTUD7B promotes PAAD progression at least in part by modulating mitochondrial bioenergetics and redox balance, thereby influencing both tumour cell fitness and the surrounding microenvironment. The results of the gene set enrichment analysis (GSEA) shown in Figure 7F further corroborate this notion, revealing enrichment of pathways related to transmembrane transport, proton‐coupled energy conversion, electron transport chain activity and NADH dehydrogenase complex function following OTUD7B depletion.

**FIGURE 7:**
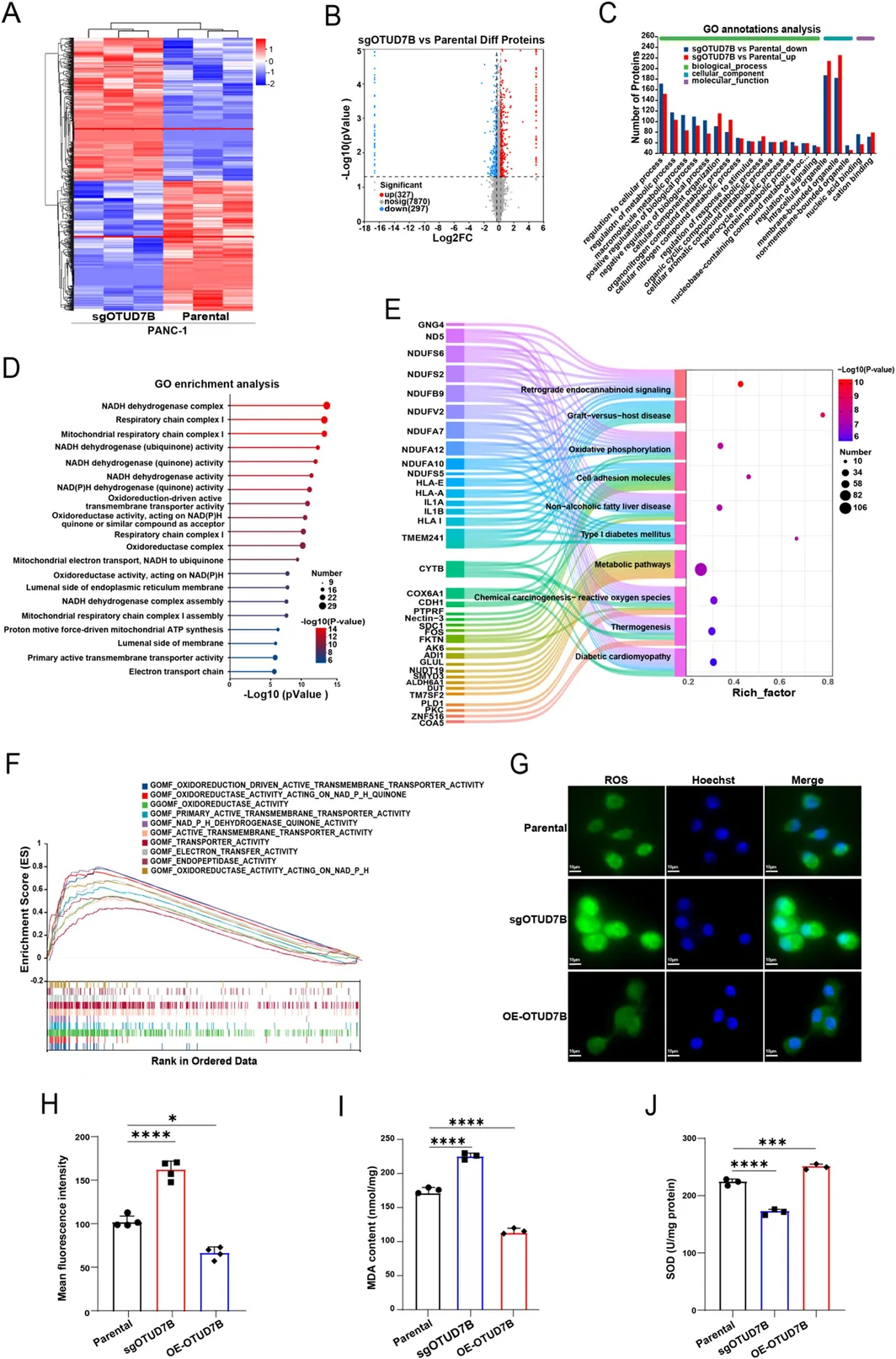
Proteomic remodelling and pathway changes after OTUD7B knockout in PAAD cells. (A) Heatmap showing global protein expression differences between OTUD7B‐knockout cells and control cells. (B) Volcano plot identifying significantly upregulated and downregulated proteins after OTUD7B loss. (C) GO annotation summary of the differentially expressed proteins. (D) Dot plot of enriched GO terms highlighting pathways related to oxidative phosphorylation, mitochondrial function and redox regulation. (E) Sankey plot and bubble chart linking key mitochondrial proteins to enriched KEGG pathways. (F) GSEA curves showing significant enrichment of mitochondrial respiratory chain, transmembrane transport and energy metabolism–related gene sets in OTUD7B‐deficient cells. (G) Representative fluorescence microscopy images showing ROS levels in the indicated cell groups. Scale bars: 10 μm. (H) Quantification of mean ROS fluorescence intensity. MDA content (I) and SOD activity (J) were assessed in the indicated cell groups. Data are presented as mean ± SEM. The asterisks (****/***) in indicate that OTUD7B overexpression or knockdown exerts statistically significant effects on the oxidative stress‐related indicators in cancer cells, including ROS, MDA and SOD.

To validate the proteomics findings, oxidative stress‐related indicators were assessed. As shown in Figure 7G–J, loss of OTUD7B significantly exacerbated oxidative stress, as evidenced by increased MDA content (Figure 7I) and ROS accumulation (Figure 7G,H), along with a concomitant decrease in SOD activity in sgOTUD7B cells compared with parental controls (Figure 7J). In contrast, OTUD7B overexpression markedly reduced MDA levels and intracellular ROS, while restoring SOD activity. Collectively, these data demonstrate that OTUD7B attenuates oxidative stress by limiting lipid peroxidation and ROS accumulation while enhancing cellular antioxidant defence capacity.

### 3.7 OTUD7B‐Associated Immunosuppressive Microenvironment and Enrichment of Regulatory T Cells in PAAD

Finally, we investigated the relationship between OTUD7B expression and the immune landscape of PAAD. In the TCGA‐PAAD cohort, as shown in Figure 8A, OTUD7B expression correlated positively with the infiltration of M2 macrophages, resting CD4 memory T cells, resting dendritic cells and activated NK cells but was negatively correlated with the expression of several effector T‐cell subsets, which is consistent with the establishment of an immunosuppressive milieu. The correlation matrix between OTUD7B and a comprehensive panel of immunomodulatory genes, including costimulatory and coinhibitory receptors, chemokines and their receptors, is shown in Figure 8B; most of these molecules are positively associated with OTUD7B, suggesting that OTUD7B is embedded within an extensive immune regulatory network. Representative scatterplots further confirmed strong positive correlations between OTUD7B expression and key immunosuppressive cell populations, including M2 macrophages, resting memory CD4^+^ T cells, and dendritic cells (Figure 8C). Proteomics data from the constructed cell models were analysed using the CIBERSORT algorithm to assess immune cell composition. As shown in Figure 8D, OTUD7B depletion markedly altered the immune landscape compared with parental controls. Specifically, activated NK cells, γδT cells, and Tregs were increased, whereas B cells (memory and naïve), CD8^+^T cells, and neutrophils showed a decreasing trend (Figure 8D,E). Consistently, multiplex immunofluorescence staining demonstrated that OTUD7B expression was positively associated with the infiltration of CD4^+^ T cells and FOXP3^+^ regulatory T cells (Figure 8F). Tumours with high OTUD7B expression exhibited markedly increased FOXP3^+^ and FOXP3^+^/CD4^+^ signals, whereas those with low OTUD7B expression showed reduced infiltration of these cell populations (Figure 8G,H). Moreover, OTUD7B expression was positively correlated with FOXP3 fluorescence intensity (Figure 8I). Collectively, these findings indicate that OTUD7B loss reshapes immune‐related signatures, supporting its role in regulating the tumour immune microenvironment.

**FIGURE 8:**
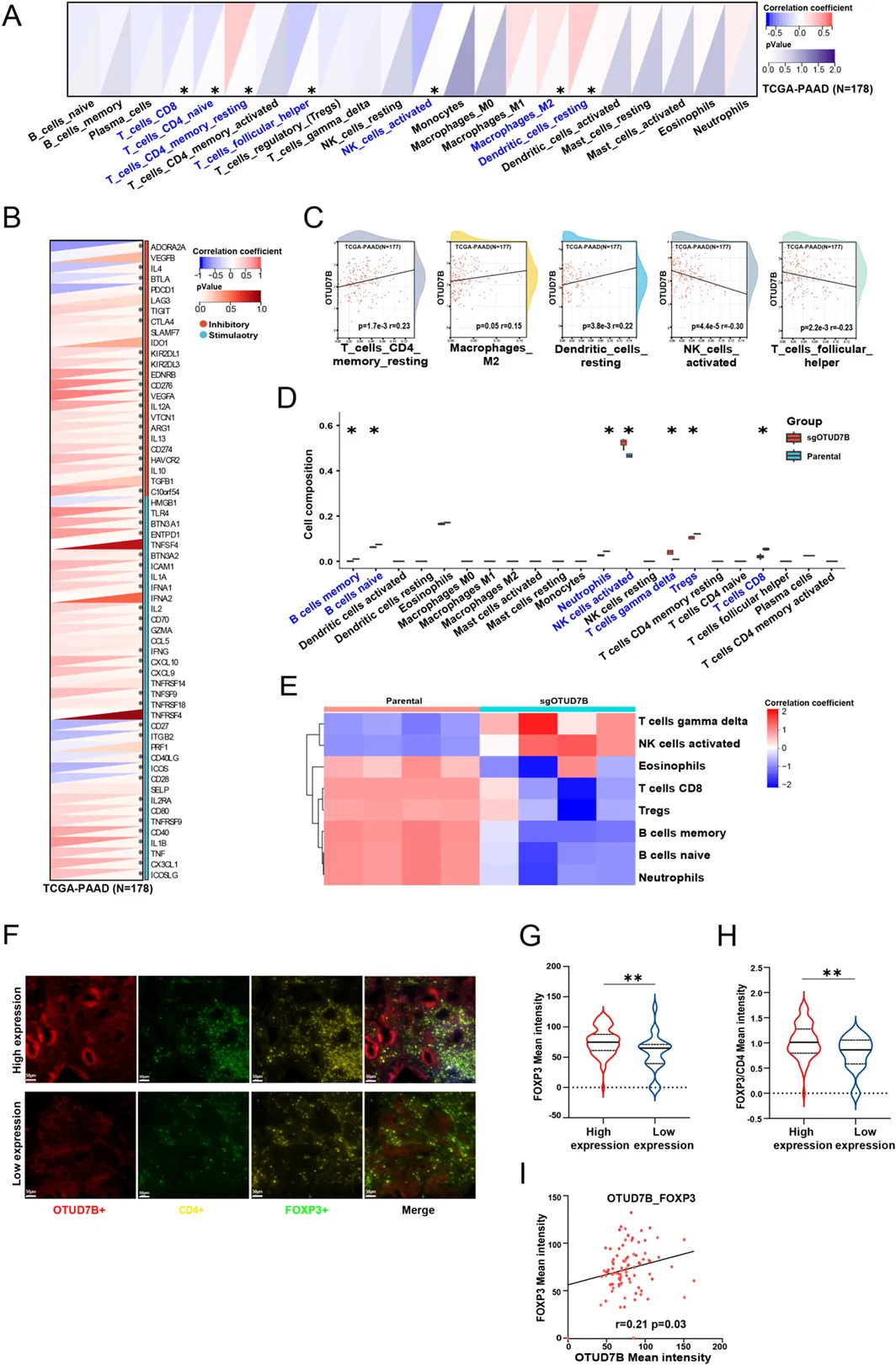
OTUD7B expression is associated with an immunosuppressive microenvironment in PAAD. (A) Correlations between OTUD7B expression and the infiltration of major immune cell subsets in the TCGA‐PAAD cohort. (B) Heatmap showing correlations between OTUD7B and immunomodulatory genes, including stimulatory and inhibitory checkpoints. (C) Representative scatterplots illustrating the relationships between OTUD7B expression and selected immune cell populations. (D) Boxplot comparison of the relative abundance of tumour‐infiltrating immune cell subtypes estimated by the CIBERSORT algorithm in sgOTUD7B versus parental tumours. (E) Correlation heatmap of significantly altered immune cell subtypes between sgOTUD7B and parental tumours. (F) Multiplex immunofluorescence staining of OTUD7B (red), CD4 (yellow) and FOXP3 (green) in low versus high OTUD7B expression groups. Scale bars: 50 μm. (G) Violin plot comparing FOXP3 fluorescence intensity between low and high OTUD7B expression groups. (H) Violin plot showing FOXP3/CD4 ratios in low versus high OTUD7B expression groups. (I) Scatterplot depicting the positive correlation between OTUD7B and FOXP3 signal intensities. The asterisks (*) in Figures 8A and 8D indicate that OTUD7B expression significantly correlated with the infiltration of indicated immune cell types both in TCGA‐PAAD groups and in cell groups. The asterisks (**) in Figures 8G and 8H indicate that OTUD7B expression was significantly correlated with FOXP3+/CD4+ T cell infiltration, supporting OTUD7B's role in regulating tumorimmunal microenvironment.

## 4 Discussion

This study integrates single‐cell transcriptomics, coexpression network analysis, nonnegative matrix factorization, proteomics, and clinical data to identify OTUD7B as a central node that links epithelial remodelling, metabolic reprogramming, and an immunosuppressive microenvironment in PAAD. Our findings suggest that the PAAD microenvironment is not simply characterized by reduced immune infiltration but is actively shaped by an OTUD7B‐high epithelial subpopulation that drives a coupled ‘metabolism–stroma–immunity’ reprogramming process, providing a plausible explanation for the tumour's high degree of aggressiveness, poor prognosis, and limited response to immunotherapy.

At the cellular and transcriptional levels, we observed marked expansion of epithelial cells, fibroblasts, and macrophages with highly active ligand–receptor communication, along with reduced effector T cells and NK cells, which is consistent with a shift from an immune‐activated state to an immune‐suppressive state. OTUD7B emerged as a hub gene within epithelial–ECM coexpression modules and marked an early‐stage epithelial subset with enhanced glycosylation and other metabolic programs, suggesting a role in tumour initiation and early evolution [19]. Clinically, OTUD7B is robustly overexpressed in PAAD, particularly in high‐grade, metastatic, and advanced‐stage tumours, and high expression is consistently associated with poor overall survival, supporting its utility as a prognostic biomarker and potential component of risk stratification models [20].

Mechanistically, quantitative proteomics revealed that OTUD7B knockout induces broad proteome remodelling, prominently affecting mitochondrial oxidative phosphorylation, electron transport, ROS metabolism, and redox homeostasis. These changes point to a key role for OTUD7B in controlling tumour bioenergetics and stress adaptation, which may, in turn, influence how tumour cells interact with and condition their microenvironment. In parallel, immune infiltration analysis and multiplex immunofluorescence demonstrated that OTUD7B expression is closely associated with enrichment of M2 macrophages and CD4^+^FOXP3^+^ regulatory T cells, as well as increased expression of multiple immunomodulatory molecules, outlining an OTUD7B–Treg–immunosuppressive axis that may underlie resistance to immune checkpoint blockade in PAAD.

Despite its strengths, this work is largely retrospective and lacks systematic in vivo validation and direct targeting of OTUD7B. Future studies should identify OTUD7B substrates and signalling partners in PAAD, delineate its cell type–specific functions within spatially resolved microenvironments, and test whether pharmacologic or genetic inhibition of OTUD7B can remodel tumour metabolism and relieve immune suppression. Such efforts may help establish OTUD7B as not only a biomarker but also a tractable therapeutic vulnerability to enhance immunotherapy efficacy in PAAD.

## Author Contributions

**Jing‐Wang:** data curation, visualization. **Xiao‐Li Liu:** writing – original draft, supervision, conceptualization. **Shan‐Yu Zhao:** validation, writing – review and editing. **Lu‐Li:** software, formal analysis. **Ming‐Hui Zhang:** funding acquisition, resources, conceptualization.

## Funding

This work was supported by grants from the Natural Science Foundation of Ningxia Medical University (XZ2022023 and XT2025039) and the Natural Science Foundation of Ningxia Province (2025AAC050079).

## Conflicts of Interest

The authors declare no conflicts of interest.

## Data Availability

The datasets used and/or analysed during the current study are available from the corresponding author upon reasonable request.
